# 2-(3,4-Dimethyl-5,5-dioxo-2*H*,4*H*-pyrazolo­[4,3-*c*][1,2]benzothia­zin-2-yl)-1-(4-meth­oxy­phen­yl)ethanone

**DOI:** 10.1107/S1600536812001286

**Published:** 2012-01-18

**Authors:** Sana Aslam, Hamid Latif Siddiqui, Matloob Ahmad, Tanvir Hussain, Masood Parvez

**Affiliations:** a Institute of Chemistry, University of the Punjab, Lahore 54590, Pakistan; b Chemistry Department, Govt. College University, Faisalabad, Pakistan; c Department of Chemistry, The University of Calgary, 2500 University Drive NW, Calgary, Alberta, Canada T2N 1N4

## Abstract

In the title mol­ecule, C_20_H_19_N_3_O_4_S, the heterocyclic thia­zine ring adopts a half-chair conformation with the S and N atoms displaced by 0.492 (6) and 0.199 (6) Å, respectively, on opposite sides from the mean plane formed by the remaining ring atoms. The ethanone group lies at an angle of 9.4 (2)° with respect to the benzene ring, which lies almost perpendicular to the pyrazole ring, with a dihedral between the two planes of 78.07 (9)°. In the crystal, mol­ecules are linked by weak C—H⋯O hydrogen bonds.

## Related literature

For the biological activity of pyrazoles, see: Farag *et al.* (2008 ▶); Ciciani *et al.* (2008 ▶); Cunico *et al.* (2006 ▶); Ahmad *et al.* (2010 ▶). For a related structure, see: Siddiqui *et al.* (2008 ▶).
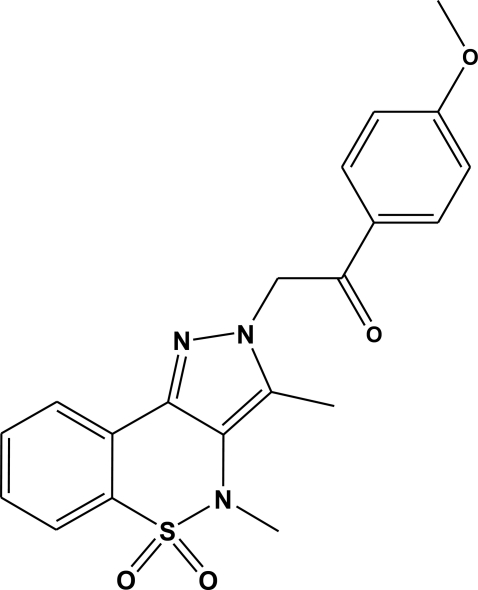


## Experimental

### 

#### Crystal data

C_20_H_19_N_3_O_4_S
*M* *_r_* = 397.44
Monoclinic, 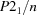

*a* = 13.862 (5) Å
*b* = 8.079 (2) Å
*c* = 17.748 (7) Å
β = 108.372 (18)°
*V* = 1886.3 (11) Å^3^
*Z* = 4
Mo *K*α radiation
μ = 0.20 mm^−1^
*T* = 173 K
0.14 × 0.09 × 0.07 mm

#### Data collection

Nonius KappaCCD diffractometer
Absorption correction: multi-scan (*SORTAV*; Blessing, 1997 ▶) *T*_min_ = 0.972, *T*_max_ = 0.986
7270 measured reflections
4221 independent reflections
3206 reflections with *I* > 2σ(*I*)
*R*_int_ = 0.046

#### Refinement

*R*[*F* ^2^ > 2σ(*F* ^2^)] = 0.063
*wR*(*F* ^2^) = 0.132
*S* = 1.15
4221 reflections
256 parameters
H-atom parameters constrained
Δρ_max_ = 0.27 e Å^−3^
Δρ_min_ = −0.34 e Å^−3^

### 

Data collection: *COLLECT* (Hooft, 1998 ▶); cell refinement: *DENZO* (Otwinowski & Minor, 1997 ▶); data reduction: *SCALEPACK* (Otwinowski & Minor, 1997 ▶); program(s) used to solve structure: *SHELXS97* (Sheldrick, 2008 ▶); program(s) used to refine structure: *SHELXL97* (Sheldrick, 2008 ▶); molecular graphics: *ORTEP-3 for Windows* (Farrugia, 1997 ▶); software used to prepare material for publication: *SHELXL97*.

## Supplementary Material

Crystal structure: contains datablock(s) global, I. DOI: 10.1107/S1600536812001286/lx2223sup1.cif

Structure factors: contains datablock(s) I. DOI: 10.1107/S1600536812001286/lx2223Isup2.hkl

Supplementary material file. DOI: 10.1107/S1600536812001286/lx2223Isup3.cml

Additional supplementary materials:  crystallographic information; 3D view; checkCIF report

## Figures and Tables

**Table 1:** Hydrogen-bond geometry (Å, °)

*D*—H⋯*A*	*D*—H	H⋯*A*	*D*⋯*A*	*D*—H⋯*A*
C1—H1⋯O1^i^	0.95	2.34	3.216 (3)	154
C4—H4⋯O4^ii^	0.95	2.53	3.435 (4)	159
C9—H9*A*⋯O3^iii^	0.98	2.54	3.335 (4)	138
C11—H11*C*⋯O2^iv^	0.98	2.56	3.498 (4)	161
C12—H12*B*⋯O2^iv^	0.99	2.33	3.309 (4)	170

Symmetry codes: (i) 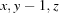; (ii) 
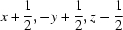
; (iii) 
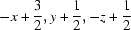
; (iv) 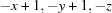.

**Fig. 1.**
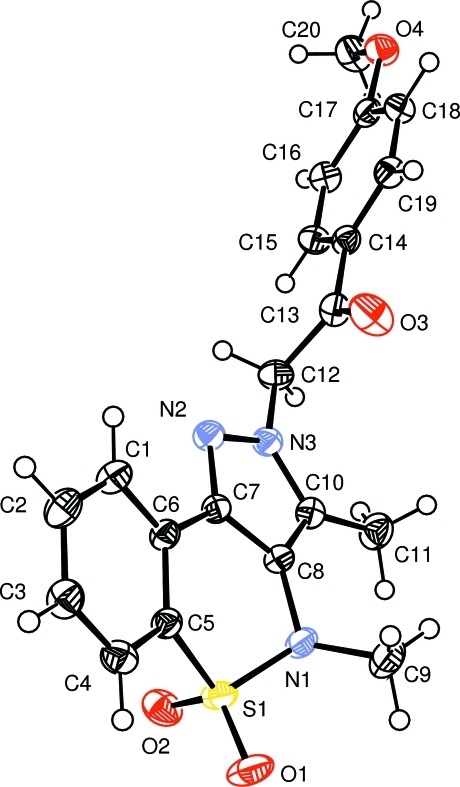
The molecular structure of the title compound with the atom numbering scheme. Displacement ellipsoids are drawn at the 30% probability level. H atoms are presented as small spheres of arbitrary radius.

**Fig. 2.**
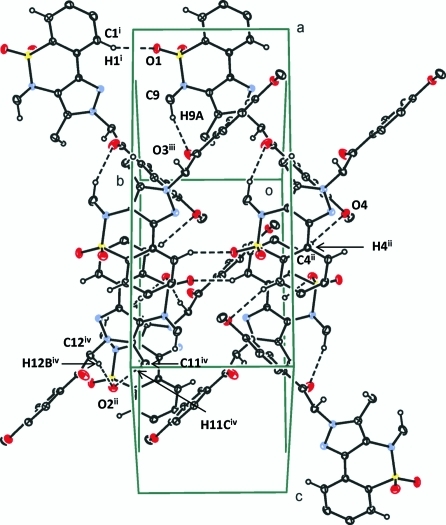
A view of the C—-H···O hydrogen bonds (dotted lines) in the crystal structure of the title compound. H atoms non-participating in hydrogen- bonding were omitted for clarity. [Symmetry codes: (i) x, y + 1, z; (ii) - x + 1/2, y + 1/2, - z + 1/2; (iii) - x + 3/2, y + 1/2, - z + 1/2; (iv) x - 1/2, y - 1/2, z + 1/2.]

## supplementary crystallographic information

### Comment

Both benzothiazines and pyrazoles are known as versatile biologically active heterocyclic nuclei. Pyrazoles are found to be cytotoxic agents (Ciciani *et al.*, 2008), anti–tumor (Farag *et al.*, 2008), anti–malarial (Cunico *et al.*, 2006), *etc*. In continuation to our research interests in biologically active molecules (Ahmad *et al.*, 2010), we have fused both of these heterocycles and herein report the synthesis and crystal structure of the title compound.

The bond distances and angles in the title compound (Fig. 1) agree very well with the corresponding bond distances and angles reported in closely related compounds (Siddiqui *et al.*, 2008). The heterocyclic thiazine ring adopts a half chair conformation with the atoms S1 and N1 displaced by 0.492 (5) and 0.199 (5) Å, respectively, on the opposite sides from the mean plane formed by the remaining ring atoms. The ethanone group O3/C12/C13/C14 is oriented at 9.4 (2)° with the benzene ring (C14–C19) which forms a dihedral angle 78.07 (9)° with the pyrazolyl ring (N2/N3/C7/C8/C10). The crystal packing (Fig. 2) is stabilized by weak intermolecular C—H···O hydrogen bonds (see, Table 1).

### Experimental

Equimolar quantities of 3,4–dimethyl–2,4–dihydropyrazolo[4,3–*c*][1,2] benzothiazine 5,5–dioxide (1.0 g, 4.01 mmol) and *p*–methoxyphenacyl bromide (0.92 g, 4.01 mmol) were dissolved in acetonitrile (20 ml) followed by the addition of equimolar K_2_CO_3_ (0.55 g, 4.01 mmol). The mixture was subjected to reflux for 7 h. The completion of reaction was monitored with the help of TLC. The precipitates of the title compound formed were collected and washed with methanol. The crystals suitable for X–ray crystallographic analysis were grown from a solution of CHCl_3_:MeOH in 1:1 ratio.

### Refinement

All H atoms were positioned geometrically and refined using a riding model, with C—H = 0.95, 0.98 and 0.99 Å, for aryl, methyl and methylene H–atoms, respectively. The *U*_iso_(H) were allowed at 1.5*U*_eq_(C methyl) or 1.2*U*_eq_(C non–methyl).

### Crystal data

**Table:**

C_20_H_19_N_3_O_4_S	*F*(000) = 832
*M**_r_* = 397.44	*D*_x_ = 1.399 Mg m^−^^3^
Monoclinic, *P*2_1_/*n*	Mo *K*α radiation, λ = 0.71073 Å
Hall symbol: -P 2yn	Cell parameters from 7270 reflections
*a* = 13.862 (5) Å	θ = 1.0–27.5°
*b* = 8.079 (2) Å	µ = 0.20 mm^−^^1^
*c* = 17.748 (7) Å	*T* = 173 K
β = 108.372 (18)°	Block, colorless
*V* = 1886.3 (11) Å^3^	0.14 × 0.09 × 0.07 mm
*Z* = 4	

### Data collection

**Table:**

Nonius KappaCCD diffractometer	4221 independent reflections
Radiation source: fine-focus sealed tube	3206 reflections with *I* > 2σ(*I*)
graphite	*R*_int_ = 0.046
ω and φ scans	θ_max_ = 27.5°, θ_min_ = 2.8°
Absorption correction: multi-scan (*SORTAV*; Blessing, 1997)	*h* = −17→17
*T*_min_ = 0.972, *T*_max_ = 0.986	*k* = −9→10
7270 measured reflections	*l* = −22→22

### Refinement

**Table:**

Refinement on *F*^2^	Primary atom site location: structure-invariant direct methods
Least-squares matrix: full	Secondary atom site location: difference Fourier map
*R*[*F*^2^ > 2σ(*F*^2^)] = 0.063	Hydrogen site location: difference Fourier map
*wR*(*F*^2^) = 0.132	H-atom parameters constrained
*S* = 1.15	*w* = 1/[σ^2^(*F*_o_^2^) + (0.0125*P*)^2^ + 3.1849*P*] where *P* = (*F*_o_^2^ + 2*F*_c_^2^)/3
4221 reflections	(Δ/σ)_max_ < 0.001
256 parameters	Δρ_max_ = 0.27 e Å^−^^3^
0 restraints	Δρ_min_ = −0.34 e Å^−^^3^

### Special details

**Table:**

Geometry. All e.s.d.'s (except the e.s.d. in the dihedral angle between two l.s. planes) are estimated using the full covariance matrix. The cell e.s.d.'s are taken into account individually in the estimation of e.s.d.'s in distances, angles and torsion angles; correlations between e.s.d.'s in cell parameters are only used when they are defined by crystal symmetry. An approximate (isotropic) treatment of cell e.s.d.'s is used for estimating e.s.d.'s involving l.s. planes.
Refinement. Refinement of *F*^2^ against ALL reflections. The weighted *R*-factor *wR* and goodness of fit *S* are based on *F*^2^, conventional *R*-factors *R* are based on *F*, with *F* set to zero for negative *F*^2^. The threshold expression of *F*^2^ > σ(*F*^2^) is used only for calculating *R*-factors(gt) *etc*. and is not relevant to the choice of reflections for refinement. *R*-factors based on *F*^2^ are statistically about twice as large as those based on *F*, and *R*- factors based on ALL data will be even larger.

### Fractional atomic coordinates and isotropic or equivalent isotropic displacement parameters (Å^2^)

**Table:**

	*x*	*y*	*z*	*U*_iso_*/*U*_eq_	
S1	0.74841 (6)	0.70621 (8)	−0.04740 (5)	0.02818 (18)	
O1	0.80588 (18)	0.8557 (2)	−0.03966 (15)	0.0421 (6)	
O2	0.65575 (16)	0.6908 (3)	−0.11268 (13)	0.0364 (5)	
O3	0.69947 (17)	0.1379 (3)	0.20986 (14)	0.0450 (6)	
O4	0.42507 (16)	−0.3992 (2)	0.30314 (12)	0.0328 (5)	
N1	0.71869 (18)	0.6790 (3)	0.03472 (15)	0.0278 (5)	
N2	0.68248 (17)	0.2335 (3)	0.02702 (14)	0.0249 (5)	
N3	0.61920 (17)	0.2872 (3)	0.06695 (14)	0.0247 (5)	
C1	0.8567 (2)	0.2439 (3)	−0.04096 (18)	0.0285 (6)	
H1	0.8446	0.1375	−0.0230	0.034*	
C2	0.9282 (2)	0.2633 (4)	−0.08003 (19)	0.0318 (7)	
H2	0.9630	0.1689	−0.0903	0.038*	
C3	0.9497 (2)	0.4180 (4)	−0.10439 (18)	0.0305 (7)	
H3	0.9994	0.4292	−0.1306	0.037*	
C4	0.8986 (2)	0.5564 (4)	−0.09057 (17)	0.0290 (6)	
H4	0.9138	0.6633	−0.1061	0.035*	
C5	0.8247 (2)	0.5358 (3)	−0.05350 (16)	0.0225 (6)	
C6	0.8025 (2)	0.3805 (3)	−0.02802 (16)	0.0226 (6)	
C7	0.7262 (2)	0.3726 (3)	0.01260 (16)	0.0222 (5)	
C8	0.6893 (2)	0.5120 (3)	0.04242 (16)	0.0238 (6)	
C9	0.7789 (3)	0.7683 (4)	0.1072 (2)	0.0394 (8)	
H9A	0.7481	0.7517	0.1493	0.059*	
H9B	0.7799	0.8867	0.0956	0.059*	
H9C	0.8486	0.7255	0.1248	0.059*	
C10	0.6204 (2)	0.4540 (3)	0.07777 (17)	0.0248 (6)	
C11	0.5603 (2)	0.5422 (4)	0.12170 (19)	0.0353 (7)	
H11A	0.5545	0.6594	0.1068	0.053*	
H11B	0.5947	0.5319	0.1789	0.053*	
H11C	0.4923	0.4932	0.1083	0.053*	
C12	0.5584 (2)	0.1665 (4)	0.09241 (18)	0.0287 (6)	
H12A	0.5394	0.0759	0.0529	0.034*	
H12B	0.4949	0.2199	0.0943	0.034*	
C13	0.6139 (2)	0.0936 (4)	0.17351 (17)	0.0266 (6)	
C14	0.5596 (2)	−0.0342 (3)	0.20525 (16)	0.0235 (6)	
C15	0.4571 (2)	−0.0723 (3)	0.16869 (17)	0.0259 (6)	
H15	0.4197	−0.0147	0.1220	0.031*	
C16	0.4092 (2)	−0.1932 (3)	0.19962 (17)	0.0260 (6)	
H16	0.3393	−0.2170	0.1747	0.031*	
C17	0.4642 (2)	−0.2790 (3)	0.26725 (16)	0.0248 (6)	
C18	0.5671 (2)	−0.2441 (3)	0.30382 (17)	0.0276 (6)	
H18	0.6048	−0.3039	0.3498	0.033*	
C19	0.6139 (2)	−0.1229 (3)	0.27307 (17)	0.0257 (6)	
H19	0.6838	−0.0993	0.2982	0.031*	
C20	0.3188 (2)	−0.4360 (4)	0.2697 (2)	0.0394 (8)	
H20A	0.2989	−0.5161	0.3035	0.059*	
H20B	0.3057	−0.4829	0.2164	0.059*	
H20C	0.2792	−0.3342	0.2662	0.059*	

### Atomic displacement parameters (Å^2^)

**Table:**

	*U*^11^	*U*^22^	*U*^33^	*U*^12^	*U*^13^	*U*^23^
S1	0.0339 (4)	0.0189 (3)	0.0368 (4)	0.0020 (3)	0.0185 (3)	0.0031 (3)
O1	0.0553 (15)	0.0190 (10)	0.0655 (17)	−0.0036 (10)	0.0381 (13)	0.0001 (10)
O2	0.0346 (12)	0.0403 (12)	0.0350 (12)	0.0117 (10)	0.0121 (10)	0.0103 (10)
O3	0.0344 (13)	0.0554 (15)	0.0397 (13)	−0.0191 (11)	0.0039 (10)	0.0091 (11)
O4	0.0365 (12)	0.0297 (11)	0.0334 (12)	−0.0089 (9)	0.0127 (10)	0.0030 (9)
N1	0.0366 (14)	0.0194 (11)	0.0333 (14)	−0.0045 (10)	0.0192 (11)	−0.0043 (10)
N2	0.0261 (12)	0.0233 (11)	0.0282 (13)	−0.0023 (9)	0.0128 (10)	0.0000 (9)
N3	0.0280 (12)	0.0231 (11)	0.0255 (12)	−0.0010 (9)	0.0120 (10)	0.0012 (9)
C1	0.0302 (15)	0.0218 (14)	0.0339 (16)	−0.0019 (11)	0.0106 (13)	−0.0032 (12)
C2	0.0284 (15)	0.0289 (15)	0.0399 (17)	0.0046 (12)	0.0133 (13)	−0.0059 (13)
C3	0.0276 (15)	0.0327 (15)	0.0354 (17)	−0.0001 (12)	0.0157 (13)	−0.0026 (13)
C4	0.0284 (15)	0.0294 (15)	0.0293 (15)	−0.0024 (12)	0.0092 (13)	0.0034 (12)
C5	0.0238 (14)	0.0204 (13)	0.0243 (14)	0.0013 (10)	0.0088 (11)	−0.0010 (10)
C6	0.0228 (13)	0.0207 (13)	0.0242 (14)	−0.0026 (10)	0.0073 (11)	−0.0028 (10)
C7	0.0245 (14)	0.0203 (13)	0.0221 (13)	−0.0030 (10)	0.0078 (11)	−0.0008 (10)
C8	0.0269 (14)	0.0193 (13)	0.0247 (14)	−0.0009 (11)	0.0075 (12)	−0.0006 (10)
C9	0.047 (2)	0.0304 (16)	0.0435 (19)	−0.0080 (14)	0.0187 (16)	−0.0124 (14)
C10	0.0248 (14)	0.0254 (13)	0.0240 (14)	0.0004 (11)	0.0075 (12)	−0.0017 (11)
C11	0.0389 (18)	0.0334 (16)	0.0387 (18)	−0.0004 (13)	0.0197 (15)	−0.0057 (14)
C12	0.0277 (15)	0.0262 (14)	0.0339 (16)	−0.0051 (12)	0.0122 (13)	0.0029 (12)
C13	0.0248 (15)	0.0292 (14)	0.0273 (15)	−0.0037 (12)	0.0105 (12)	−0.0029 (12)
C14	0.0250 (14)	0.0225 (13)	0.0256 (14)	−0.0008 (11)	0.0116 (12)	−0.0019 (11)
C15	0.0245 (14)	0.0261 (14)	0.0278 (15)	0.0006 (11)	0.0094 (12)	0.0039 (11)
C16	0.0199 (13)	0.0287 (14)	0.0302 (15)	−0.0011 (11)	0.0092 (11)	0.0001 (12)
C17	0.0297 (15)	0.0207 (13)	0.0269 (14)	−0.0039 (11)	0.0131 (12)	−0.0039 (11)
C18	0.0307 (15)	0.0268 (14)	0.0245 (14)	0.0027 (12)	0.0074 (12)	0.0027 (11)
C19	0.0217 (14)	0.0264 (14)	0.0271 (14)	0.0003 (11)	0.0051 (12)	−0.0018 (11)
C20	0.0378 (18)	0.0431 (19)	0.0376 (18)	−0.0157 (15)	0.0124 (15)	0.0034 (15)

### Geometric parameters (Å, °)

**Table:**

S1—O1	1.429 (2)	C8—C10	1.380 (4)
S1—O2	1.439 (2)	C9—H9A	0.9800
S1—N1	1.650 (3)	C9—H9B	0.9800
S1—C5	1.760 (3)	C9—H9C	0.9800
O3—C13	1.212 (3)	C10—C11	1.489 (4)
O4—C17	1.365 (3)	C11—H11A	0.9800
O4—C20	1.435 (4)	C11—H11B	0.9800
N1—C8	1.428 (3)	C11—H11C	0.9800
N1—C9	1.482 (4)	C12—C13	1.521 (4)
N2—C7	1.340 (3)	C12—H12A	0.9900
N2—N3	1.361 (3)	C12—H12B	0.9900
N3—C10	1.360 (3)	C13—C14	1.489 (4)
N3—C12	1.451 (3)	C14—C15	1.397 (4)
C1—C2	1.386 (4)	C14—C19	1.399 (4)
C1—C6	1.394 (4)	C15—C16	1.387 (4)
C1—H1	0.9500	C15—H15	0.9500
C2—C3	1.385 (4)	C16—C17	1.388 (4)
C2—H2	0.9500	C16—H16	0.9500
C3—C4	1.386 (4)	C17—C18	1.397 (4)
C3—H3	0.9500	C18—C19	1.378 (4)
C4—C5	1.391 (4)	C18—H18	0.9500
C4—H4	0.9500	C19—H19	0.9500
C5—C6	1.400 (4)	C20—H20A	0.9800
C6—C7	1.457 (4)	C20—H20B	0.9800
C7—C8	1.407 (4)	C20—H20C	0.9800
			
O1—S1—O2	118.68 (14)	H9B—C9—H9C	109.5
O1—S1—N1	108.34 (13)	N3—C10—C8	104.5 (2)
O2—S1—N1	106.97 (13)	N3—C10—C11	124.4 (3)
O1—S1—C5	109.83 (13)	C8—C10—C11	131.1 (3)
O2—S1—C5	106.49 (13)	C10—C11—H11A	109.5
N1—S1—C5	105.80 (13)	C10—C11—H11B	109.5
C17—O4—C20	117.5 (2)	H11A—C11—H11B	109.5
C8—N1—C9	118.4 (2)	C10—C11—H11C	109.5
C8—N1—S1	111.63 (18)	H11A—C11—H11C	109.5
C9—N1—S1	118.2 (2)	H11B—C11—H11C	109.5
C7—N2—N3	103.7 (2)	N3—C12—C13	112.6 (2)
C10—N3—N2	114.1 (2)	N3—C12—H12A	109.1
C10—N3—C12	127.2 (2)	C13—C12—H12A	109.1
N2—N3—C12	118.7 (2)	N3—C12—H12B	109.1
C2—C1—C6	120.0 (3)	C13—C12—H12B	109.1
C2—C1—H1	120.0	H12A—C12—H12B	107.8
C6—C1—H1	120.0	O3—C13—C14	122.0 (3)
C3—C2—C1	121.1 (3)	O3—C13—C12	120.5 (3)
C3—C2—H2	119.5	C14—C13—C12	117.5 (2)
C1—C2—H2	119.5	C15—C14—C19	118.6 (2)
C2—C3—C4	120.1 (3)	C15—C14—C13	122.6 (2)
C2—C3—H3	120.0	C19—C14—C13	118.8 (2)
C4—C3—H3	120.0	C16—C15—C14	121.0 (3)
C3—C4—C5	118.7 (3)	C16—C15—H15	119.5
C3—C4—H4	120.7	C14—C15—H15	119.5
C5—C4—H4	120.7	C15—C16—C17	119.5 (3)
C4—C5—C6	122.0 (3)	C15—C16—H16	120.2
C4—C5—S1	118.8 (2)	C17—C16—H16	120.2
C6—C5—S1	118.9 (2)	O4—C17—C16	124.6 (3)
C1—C6—C5	118.1 (2)	O4—C17—C18	115.2 (2)
C1—C6—C7	124.0 (2)	C16—C17—C18	120.2 (3)
C5—C6—C7	117.8 (2)	C19—C18—C17	119.9 (3)
N2—C7—C8	111.0 (2)	C19—C18—H18	120.1
N2—C7—C6	125.0 (2)	C17—C18—H18	120.1
C8—C7—C6	123.9 (2)	C18—C19—C14	120.8 (3)
C10—C8—C7	106.6 (2)	C18—C19—H19	119.6
C10—C8—N1	128.6 (2)	C14—C19—H19	119.6
C7—C8—N1	124.8 (2)	O4—C20—H20A	109.5
N1—C9—H9A	109.5	O4—C20—H20B	109.5
N1—C9—H9B	109.5	H20A—C20—H20B	109.5
H9A—C9—H9B	109.5	O4—C20—H20C	109.5
N1—C9—H9C	109.5	H20A—C20—H20C	109.5
H9A—C9—H9C	109.5	H20B—C20—H20C	109.5
			
O1—S1—N1—C8	−162.2 (2)	C6—C7—C8—N1	2.2 (4)
O2—S1—N1—C8	68.8 (2)	C9—N1—C8—C10	69.1 (4)
C5—S1—N1—C8	−44.4 (2)	S1—N1—C8—C10	−148.3 (3)
O1—S1—N1—C9	−19.6 (3)	C9—N1—C8—C7	−112.1 (3)
O2—S1—N1—C9	−148.6 (2)	S1—N1—C8—C7	30.4 (4)
C5—S1—N1—C9	98.2 (2)	N2—N3—C10—C8	−0.1 (3)
C7—N2—N3—C10	0.6 (3)	C12—N3—C10—C8	−179.5 (3)
C7—N2—N3—C12	180.0 (2)	N2—N3—C10—C11	−178.1 (3)
C6—C1—C2—C3	2.2 (5)	C12—N3—C10—C11	2.5 (5)
C1—C2—C3—C4	−0.7 (5)	C7—C8—C10—N3	−0.4 (3)
C2—C3—C4—C5	−1.3 (4)	N1—C8—C10—N3	178.5 (3)
C3—C4—C5—C6	1.7 (4)	C7—C8—C10—C11	177.4 (3)
C3—C4—C5—S1	−171.8 (2)	N1—C8—C10—C11	−3.7 (5)
O1—S1—C5—C4	−33.1 (3)	C10—N3—C12—C13	−92.9 (3)
O2—S1—C5—C4	96.6 (2)	N2—N3—C12—C13	87.7 (3)
N1—S1—C5—C4	−149.8 (2)	N3—C12—C13—O3	0.4 (4)
O1—S1—C5—C6	153.1 (2)	N3—C12—C13—C14	−179.0 (2)
O2—S1—C5—C6	−77.2 (2)	O3—C13—C14—C15	172.2 (3)
N1—S1—C5—C6	36.4 (3)	C12—C13—C14—C15	−8.5 (4)
C2—C1—C6—C5	−1.8 (4)	O3—C13—C14—C19	−9.7 (4)
C2—C1—C6—C7	−179.8 (3)	C12—C13—C14—C19	169.6 (3)
C4—C5—C6—C1	−0.2 (4)	C19—C14—C15—C16	1.3 (4)
S1—C5—C6—C1	173.3 (2)	C13—C14—C15—C16	179.4 (3)
C4—C5—C6—C7	178.0 (3)	C14—C15—C16—C17	−0.9 (4)
S1—C5—C6—C7	−8.5 (3)	C20—O4—C17—C16	−1.8 (4)
N3—N2—C7—C8	−0.8 (3)	C20—O4—C17—C18	177.9 (3)
N3—N2—C7—C6	178.8 (2)	C15—C16—C17—O4	179.5 (3)
C1—C6—C7—N2	−15.4 (4)	C15—C16—C17—C18	−0.1 (4)
C5—C6—C7—N2	166.6 (3)	O4—C17—C18—C19	−179.0 (2)
C1—C6—C7—C8	164.2 (3)	C16—C17—C18—C19	0.7 (4)
C5—C6—C7—C8	−13.9 (4)	C17—C18—C19—C14	−0.2 (4)
N2—C7—C8—C10	0.8 (3)	C15—C14—C19—C18	−0.7 (4)
C6—C7—C8—C10	−178.8 (3)	C13—C14—C19—C18	−179.0 (3)
N2—C7—C8—N1	−178.2 (3)		

### Hydrogen-bond geometry (Å, °)

**Table:**

*D*—H···*A*	*D*—H	H···*A*	*D*···*A*	*D*—H···*A*
C1—H1···O1^i^	0.95	2.34	3.216 (3)	154.
C4—H4···O4^ii^	0.95	2.53	3.435 (4)	159.
C9—H9A···O3^iii^	0.98	2.54	3.335 (4)	138.
C11—H11C···O2^iv^	0.98	2.56	3.498 (4)	161.
C12—H12B···O2^iv^	0.99	2.33	3.309 (4)	170.

Symmetry codes: (i) *x*, *y*−1, *z*; (ii) *x*+1/2, −*y*+1/2, *z*−1/2; (iii) −*x*+3/2, *y*+1/2, −*z*+1/2; (iv) −*x*+1, −*y*+1, −*z*.
